# Fostering Learning Goals at Work: The Interplay of Dispositional and Workplace Learning Goal Orientation and Supervisor Appraisal Behavior

**DOI:** 10.1177/00332941231198057

**Published:** 2023-08-29

**Authors:** Leonie Schelp, Tanja Bipp, Sabrina Gado, Martin Daumiller

**Affiliations:** 9190 Julius-Maximilians Universität Würzburg, Würzburg, Germany; 9144 Heidelberg University, Heidelberg, Germany; 9190 Julius-Maximilians Universität Würzburg, Würzburg, Germany; 26522 University of Augsburg, Augsburg, Germany

**Keywords:** Workplace goal orientation, appraisal, personnel development, learning

## Abstract

A workplace that emphasizes personal learning and task mastery fosters employee development and performance. However, it is yet unclear which specific factors support such a learning goal-oriented workplace. Based on research in the educational domain, we investigated the reciprocal effects of dispositional learning goal orientation, supervisor’s appraisal behavior, and a learning goal-oriented workplace. In a study with a repeated measurement design (*N* = 144 employees), we did not find support for an effect of supervisor’s appraisal behavior (operationalized by the perceived use of self-reference norms and constructive handling of errors by employees) on workplace learning goal orientation over time. However, we found that a dispositional learning goal orientation of employees supports a learning goal-oriented work environment. Furthermore, workplace learning goal orientation had a cross-lagged effect on dispositional learning goal orientation and supervisor’s appraisal behavior. By comparing our results from work to findings from the educational context, our results convey important theoretical implications about the construct of workplace goal orientation and suggest practical applications to foster a learning goal-oriented workplace in terms of personnel development and performance management.

The nature of work has changed, and “the 21st century has been marked by a focus on employee development and continuous learning to engage and retain employees” (Linderbaum & Levy, 2010, p. 1372). To stimulate learning and growth at work, we build up on Achievement Goal Theory which postulates that individuals’ motivation is not only influenced by their personal goals but also by contextual goal structures (e.g., Ames, 1992a; Anderman & Patrick, 2012). On the one hand, a (dispositional) learning goal orientation has been found to be positively associated with learning process variables or the motivation to learn at work (e.g., Chung et al., 2022). On the other hand, also the work environment has an impact on learning and development (e.g., Anderman & Patrick, 2012; Bardach et al., 2020; Chung et al., 2022). We focus in the current study on the construct of *workplace goal orientation* as a contextual variable, that targets the employees’ perceptions of the *goal structure* of their work environment (Van Dam, 2015). In line with the positive effects reported in the literature for dispositional learning goal orientation at work (e.g. Cellar et al., 2011), also a learning goal-oriented workplace is positively related to desirable behavioral and achievement outcomes (e.g., Theis & Bipp, 2020). However, little is known yet about the stability of the perception of a learning goal-oriented workplace or reciprocal relationships with dispositional goal orientation. Furthermore, given that the behavior of supervisors has been suggested to play a key role for successful learning and development of employees (e.g., Hannah & Lester, 2009), there is the need to specify if and how supervisors can promote a learning goal-oriented work environment.

Therefore, the aim of the current study is threefold. First, to derive practical implications for the work context to stimulate a learning goal orientation at work, it needs to be specified to what extent the perception of the goal structure of the work environment changes over time. Therefore, we provide insights into whether employees perceive the learning goal structure of their workplace as relatively stable or dynamic. Second, we investigate the interplay of dispositional and workplace learning goal orientation in a repeated measurement design with two time points, specifying their potential reciprocal relationships over a period of five weeks. Third, we investigate the influence of the (perceived) appraisal behavior by supervisors on workplace learning goal orientation. While findings in the educational context indicate that appraisal aspects communicated by teachers foster a learning goal-oriented environment in the classroom (e.g., Benning et al., 2019), it is not clear yet whether these findings can be transferred to the work context.

## Theoretical Background

### Dispositional Goal Orientations and Contextual Goal Structures

Originating from the educational domain, the concept of goal orientation has been successfully transferred to the work setting (e.g., Bipp et al., 2021). Goal orientation theory (VandeWalle et al., 2019) outlines the effect of goal orientations, conceptualized as individual dispositions of employees, on proximal mediators and distal outcomes in terms of organizational behavior. Within the work domain, the so-called trichotomous goal framework (separating learning, performance-approach, and performance-avoidance goals) has received broad attention and empirical support (DeShon & Gillespie, 2005). Research supports the predictive validity of goal orientations at work with regard to performance outcomes or self-regulatory behavior. For example, in their meta-analysis, Cellar and colleagues (2011) found a positive relationship between mastery (or learning) goal orientation and self-efficacy or work performance.

In contrast, the related concept of contextual goal structures in terms of workplace goal orientation has just recently gained attention in the organizational behavior literature (Van Dam, 2015). Building upon achievement goal theory, situational factors, and instructional demands are expected to influence the salience of particular goals and hence their adoption in achievement settings (e.g., Ames, 1992a; Bardach et al., 2020). In the educational domain, the construct of classroom goal structure is prominently used to describe the effect of environmental factors within learning settings (e.g., Ames, 1992b; Meece et al., 2006). Following Anderman and Patrick (2012), it “encompasses students’ subjective perceptions of the meaning of academic tasks, competence, success, and purposes for students’ engaging in schoolwork” (p. 181). Numerous studies have shown that classroom goal structures affect learning-related outcomes, for example, intrinsic motivation, self-efficacy, learning activities, or success (e.g., Urdan & Turner, 2005; Wolters, 2004). Comparable, at work, contextual goal structures refer to the individual perception of goal structures of the work environment and are expected to affect relevant work outcomes. With regard to the construct of workplace goal orientation, three types of goals have been suggested (Theis & Bipp, 2020; Van Dam, 2015): (1) workplace learning, (2) workplace performance-approach, and (3) workplace performance-avoidance goal orientation.

### Dispositional and Workplace Learning Goal Orientation

Of the three postulated dimensions of classroom goal structures (learning or mastery,^1^ performance-approach, and performance-avoidance; Midgley et al., 2000), mainly learning goal structures have been shown to be positively related to desirable outcomes such as successful learning or motivation (e.g., Urdan & Turner, 2005; Wolters, 2004). A classroom learning goal structure is characterized by norms and instructional practices which emphasize understanding, improvement, and learning (Midgley et al., 2000). Looking at the definition of a learning goal-oriented workplace (e.g., Van Dam, 2015), such a work environment also emphasizes personal growth and provides opportunities to learn for employees. In particular, success is defined as an improvement and thus implies an intraindividual reference framework (comparing the current performance with the own previous one, not to the performance of others). Also at work, a *workplace learning goal orientation* seems to be positively related to favorable outcomes. For example, individuals who perceive the work environment as learning goal-oriented report higher self-efficacy and more proactive behavior (Schelp et al., 2022; Theis & Bipp, 2020). Furthermore, positive correlations between workplace learning goal orientation and outcomes such as learning success and performance have been reported, showing incremental validity above and beyond dispositional goal orientations (e.g., Van Dam, 2015). Therefore, it seems essential for companies to acquire knowledge on how to foster a learning goal-oriented work environment. And, to fully integrate the concept into goal orientation theory (VandeWalle et al., 2019), there is a need to provide insights into the nature of workplace goal orientation in terms of stability and the interplay with dispositional goal orientation.

First, a certain stability of learning workplace goal orientation can be anticipated. This expectation is based on research findings in the educational context for the related variable of classroom goal structures. For example, Bergsmann et al. (2013) investigated the perception of a supportive (comparable to learning) goal structure of schoolchildren over a nine-month period. They found that a supportive goal structure at the beginning positively predicts the perceived goal structure nine months later (*r* = .34, *p* < .001), while pupils stayed with the same teacher and classmates during this period. However, given its definition as an environmental variable, workplace goal orientation is not supposed to be as stable over time as dispositional goal orientation. Regarding the latter, the meta-analytic finding of Payne et al. (2007) support the stability of trait learning goal orientation (sample weighted mean *r* = .66).

Accordingly, we propose the following:

H1: Workplace learning goal orientation at T1 is positively related to workplace learning goal orientation at T2.

Second, taking a look at the interplay between dispositional and workplace learning goal orientation, prior research has indicated positive correlations to a moderate degree (Theis & Bipp, 2020; Van Dam, 2015). On the one hand, such a relationship might be due to the impact of dispositional goal orientation on the perception of learning goals at work. Building upon the person-environment fit perspective (Barrick & Parks-Leduc, 2019), employees with high learning goal orientation might seek out or create a work situation or an environment that fits their disposition. Indeed, trait goal orientation has been shown to be positively related to state goal orientation (Payne et al., 2007). More specifically with regard to organizational learning, in their multilevel theory, Chadwick and Raver (2015) suggest that an employee’s goal orientation affects not only goal orientation at the group level but also the organizational goal orientation culture.

On the other hand, it is not clear if the workplace can also affect an employee’s learning goal orientation over time. Various variables have been suggested as potential determinants of trait goal orientation (e.g., personality traits; Payne et al., 2007). However, given the mainly correlational study designs, clear empirical evidence for the causal ordering among the variables in the nomological net of goal orientation is sparse (VandeWalle et al., 2019). However, research has shown that working in a particular environment can lead to changes in dispositional traits (e.g., Woods et al., 2013). With regard to goals, specifically designed interventions have also been shown to alter goal orientation (e.g., Wang et al., 2018). In their meta-analysis for the educational context, Bardach et al. (2020) showed that students indeed adopt (to a certain degree) the goals that they perceive in the learning environment. We expected that working in an environment with a strong learning goal structure that emphasizes personal development and provides opportunities to learn, might also promote the own learning goal orientation of employees. Therefore, we anticipated the following reciprocal relationships between these two related, yet distinct constructs over time.

H2a: Dispositional learning goal orientation at T1 is positively related to workplace learning goal orientation at T2.

H2b: Workplace learning goal orientation at T1 is positively related to dispositional learning goal orientation at T2.

### Supervisor’s Appraisal Behavior and Workplace Learning Goal Orientation

In search of approaches to support a workplace learning goal orientation, we looked at prior research findings in the educational domain. In this context, various studies have shown that the behavior of teachers contributes to the perception of classroom goal structures via their instructional practices (Anderman & Patrick, 2012). In particular, the TARGET framework has received a lot of attention that outlines six different instructional strategies (task, authority, recognition, grouping, evaluation, and time) that impact students’ learning, performance, and motivation (e.g., Ames, 1992a; Lüftenegger et al., 2014; Meece et al., 2006). Although the framework is well established to foster a learning goal structure in the educational domain (e.g., Cecchini et al., 2014), considerable overlap between the six suggested dimensions led to further theoretical advancements. We relied on the work done by Benning et al. (2019), who suggested four instructional dimensions based on the original TARGET model: *content* (referring to time and task dimensions), *appraisal* (including evaluation and recognition), *autonomy* (resembling the authority dimension in other models), and *social* (includes aspects of the grouping dimension).

In particular, the appraisal dimension seems promising to foster a workplace learning goal orientation. For the educational context, Ames (1992a) emphasized that “the ways in which students are evaluated is one of the most salient classroom factors that can affect student motivation” (p. 264). Teachers should, for example, focus on individual improvement and progress, make evaluations in private, and provide opportunities for learning to promote a learning goal structure and thereby facilitate positive outcomes. Further, Benning et al. (2019) found a strong effect of the appraisal dimension on a learning goal structure. More specifically, in their framework model, Benning et al. (2019) suggested three relevant facets of appraisal that foster a learning goal-oriented environment (at school): (1) self-reference norm, (2) constructive handling of errors, and (3) effort-related feedback.

First, the reference norm is comparable to a benchmark a person uses to assess and evaluate one's performance. It can be either an intrapersonal self-oriented norm or an interpersonal other-oriented norm. A self-referenced (or individual) norm in which performance is compared to one’s own previous performances is expected to support a learning goal structure in the educational context (Benning et al., 2019). In addition, Church et al. (2001) provided indirect empirical support for such an effect, as they showed that the evaluation approach displayed by teachers is indeed related to goal structures. In their study, the use of an absolute (compared to normative) standard was associated with a higher learning goal structure.

Second, regarding the handling of errors, Steuer et al. (2013) showed that there is a substantial relationship between the way a teacher handles errors in the classroom and the perceived classroom goal structure by students. Constructive error handling, characterized by error tolerance, support following errors, absence of negative reactions after errors, and encouragement to risk errors, is assumed to have positive effects on learning processes and achievement (Steuer et al., 2013).

Third, the way of giving feedback has, according to Benning et al. (2019), a large impact on students’ efforts. If teachers give effort-related, individual, and constructive feedback, students can attribute their achievements to their personal endeavors and hence perceive their environment as learning goal-oriented. Empirical support for such an effect stems from Linnenbrink (2005), who was able to manipulate the goal structure in a classroom setting through specific forms of feedback. She found that learning goals were indeed stimulated by emphasizing the importance of learning, understanding, and improvement.

Based on these promising findings about how teachers’ appraisal behavior can stimulate a learning goal-oriented classroom, the question arises if it is possible to transfer these effects to the work context. Also at work, evaluation and feedback are seen as essential elements influencing employees’ motivation, learning, and performance (e.g., Kluger & DeNisi, 1996). In a recent meta-analysis, Katz et al. (2021) provided support for the utility of feedback by shedding light on the relationship of a positive feedback environment to various important work outcomes, such as job satisfaction or well-being of employees. Or, with reference to a specific group of employees, Dickhäuser et al. (2021) found that the perception of a positive feedback culture at schools is associated with the learning goal orientation of teachers. A critical role to support learning goal orientation at work can therefore be anticipated in the (formal and informal) appraisal behavior of supervisors. For example, supervisors are an important source of feedback, and they are considered to be an essential force behind employee engagement in learning activities through the use of goal setting and evaluation (Bezuijen et al., 2010; Sonnentag et al., 2004). Feedback that focuses on the possibilities for personal improvement has been suggested to encourage the learning activities of employees (Mulder, 2013). With regard to leadership, for example, authentic or transformational leadership behavior was found to be linked to learning goals of employees (e.g., Hamstra et al., 2014; Mehmood, et al., 2016). Furthermore, Liu and Xiang (2020) showed, that coaching behaviors of supervisors encouraging exploration or guiding to learn are related to an employee’s learning goal orientation. However, is not yet known which specific supervisor appraisal behavior can contribute to a learning goal-oriented work environment.

Based on the suggested dimensionality of appraisal behavior by Benning et al. (2019), we expected that employees who perceive that their supervisor uses self-reference norms, provides effort-related feedback, and handles errors in a constructive manner, are more likely to report higher values of workplace learning goal orientation. On the one hand, such a relationship might be explained by a positive effect of the supervisor’s appraisal behavior on workplace learning goal orientation, comparable to the effect outlined above for teachers on a learning goal structure in the educational context. On the other hand, a work environment that emphasizes learning goals might also impact the appraisal behavior of supervisors in return. Given that the perceptions of a learning goal work environment encompass more than the behavior of the direct supervisor but refers to a more general perception of the possibilities to develop in an organization, the goal structure at work might also affect the evaluation and feedback provided by supervisors. Hence, we propose the following:

H3a: Supervisor’s appraisal behaviors at T1 are positively related to workplace learning goal orientation at T2.

H3b: Workplace learning goal orientation at T1 is positively related to supervisor’s appraisal behaviors at T2.

## Method

### Procedure and Participants

German employees from different companies were invited to participate in a two-phase online study by e-mail. The participants were guaranteed confidentiality and were informed that participation was voluntary and that they could withdraw at any time during the data collection. As incentives, participants got general feedback on the study results and participated in a raffle of vouchers (6 × 50 Euro). Given the non-interventional design, it was decided that a thorough approval by the local ethics committee was not necessary for this study (an initial short application did not result in the request for a full proposal in accordance with local regulations). After being informed and providing consent, participants proceeded to the questionnaire. Data were collected using two identical surveys, each consisting of two waves with a 5-week interval in between. Given that neither achievement goal nor goal orientation theory specifically encompasses the aspect of time that allows us to define a particular time frame for investigating the stability (or change) of contextual goal structures, we decided to use this interval in line with prior work in the area of goal orientation. Daumiller et al. (2023) investigated the stability of achievement goal orientations of scientific personnel with an intensive micro-longitudinal design and documented between one-third and half of variability in goal pursuit across a five-week time span. In Survey 1, taking place from May until July 2019, 240 questionnaires were filled out in the first wave. In the second wave of Survey 1, 118 of the previous participants filled in the questionnaire. Of those, 88 data sets could be merged based on a personal code (36% response rate T1-T2). To enlarge the sample size, we collected additional data in a second survey from October until December 2020. In the first wave of this survey, 109 employees provided data. Out of 103 participants who took part in the second wave of this survey, we were able to match *n* = 56 participants (51% response rate T1-T2).

Given that for one survey, data was collected before, and for the second survey during the COVID-19 pandemic in which Germany was still in a state with a lot of restrictions (e.g., mandatory home office for some occupations), we checked the two datasets for potential differences regarding our study variables using a multivariate analysis of variance (MANOVA). Since we detected no significant differences, *F*(8, 134) = 1.12, *p* = .36; we combined both datasets for the analysis which resulted in a final sample of *N* = 144 across the two time points. Respondents were, on average 34.16 years old (*SD* = 10.82), and 61.8% were female. The sample consisted of employees who worked in a variety of areas. The majority worked in the following sectors: Sciences (19.9%), health (18.8%), industry (14.6%), information technology (7.6%), law & order or state administration (6.3%), and social (5.0%). Participants indicated an average working experience of 10.82 years (*SD* = 10.66) and the mean working hours per week was 37.17 (*SD* = 9.61).

### Measures

All measures were collected at both time points in random order.

#### Workplace Learning Goal Orientation

We assessed workplace learning goal orientation with the German version (Theis & Bipp, 2020) of the workplace goal orientation measure (Van Dam, 2015). The scale encompasses five items (e.g., “I find my workplace to be a place in which people get time to learn”). The response scale ranged from 1 (*strongly disagree*) to 5 (*strongly agree*). The internal consistency for the scale was ω = .90 (McDonald Omega; McDonald, 2013) at both time points.

#### Dispositional Learning Goal Orientation

We assessed dispositional learning goal orientation with a German translation of the four-item subscale by VandeWalle (2001) that was successfully used in prior research (Theis & Bipp, 2020; e.g., “I enjoy challenging and difficult tasks at work where’ll learn new skills”). Responses were given on a 7-point Likert scale (1 = *strongly disagree*, 7 = *strongly agree*). Values for Omega ranged in the current study between .86 and .87.

#### Appraisal Facets

We assessed the three appraisal facets with an adaptation of the validated scales developed for the educational context by Benning et al. (2019). We pretested the adaption of items to the work context (targeting the work setting and supervisor, instead of the school context and teachers) in a sample of 104 university students with work experience (71 female; average age of 22.85 years; *SD =* 2.85). Participants were invited via e-mail (online version) or personally during university lectures (paper version). We asked participants to answer demographic questions and 16 adapted items of the appraisal scale by Benning et al. (2019). The original scale includes three dimensions: (1) setting of self-referenced norms, (2) giving effort-related feedback, and (3) constructive handling of errors by the teacher (Benning et al., 2019). In detail, we adapted the items to the work context and replaced “us” with “me” in the item formulation to capture the subjective perception of employees. Furthermore, we deleted seven items of the scale assessing constructive handling of errors because they also include the evaluation by and behavior of classmates because we wanted to focus on the appraisal by supervisors. As a result, we assessed the setting of self-referenced norms with three items (e.g., “My leader says the output is satisfying when it shows improvement to prior work results”), giving effort-related feedback with four items (e.g., “My supervisor says that good results in my work unit are led-back to good preparation”) and constructive handling of errors with nine items (e.g., “My supervisor assists in case of misdoing”). Responses were given on a 6-point Likert scale (1 = *strongly disagree*, 6 = *strongly agree*).

The results of the pretest indicated that we partially succeeded with the adaption of the scales for the work context. We calculated two different indices for the internal consistency of the scales: Omega and Cronbach’s α. In detail, Omega for self-reference norm was .74 (α = .72), and .85 (α = .79) for constructive handling of errors. These results are comparable to the original version (Benning et al., 2019). However, the values for the scale of effort-related feedback (ω_t_ = .62; α = .50) was unsatisfactory. Therefore, we decided to exclude this subscale from the main study. Both remaining appraisal facets reached again acceptable values for reliability estimates in the two online surveys, with Omega ranging from .73 – .85 for self-referenced norms, and .82 – .86 for constructive handling of errors.

### Analytic Strategy

To test our hypotheses, we modeled and tested a series of path models using AMOS 27.0 (Arbuckle, 2017) with maximum likelihood. In a baseline model (stability model, M1) we included the temporal stabilities of all four constructs (use of self-reference norms, constructive handling of errors, workplace, and dispositional learning goal orientation) and included the synchronous correlations among the constructs for each measurement point. In the following, we compared the fit of this model to a more complex one including the expected reciprocal effects (reciprocal model, M2). In detail, we, first, added three paths from the two appraisal facets and dispositional learning goal orientation from T1 to workplace learning goal orientation at T2 and, second, paths from workplace learning goal orientation at T1 to the two appraisal facets and dispositional learning goal orientation at T2. We compared the two models on a variety of fit indices (Hu & Bentler, 1999): χ^2^ goodness-of-fit statistic, the Normed Fit Index (NFI), the Incremental Fit Index (IFI), the Comparative Fit Index (CFI), and the Root Mean Square Error of Approximation (RMSEA).

We inspected the relationships of several demographic variables with the main study variables in our diverse sample. Age, work experience, and gender did not correlate in a consistent manner with workplace learning goal orientation or supervisor’s appraisal behavior across both measurement points, with one exception: A small, negative relationship between age (and the highly related variable of work experience) and dispositional learning goal orientation was visible at T1 and T2 (*r* = −.22).

## Results

Table 1 displays descriptive statistics of the study variables. Taking a closer look at the intercorrelations, all constructs correlated significantly over time, with retest correlations ranging from .47 (self-reference norm) to .77 (constructive handling of errors). Furthermore, the general correlational pattern among the study variables could be replicated across the two time points (with one deviation: workplace learning goal orientation did not correlate with self-reference norm at T1 but showed a positive relationship at T2).

**Table 1.** Means (M), Standard Deviations (SD), Reliabilities, and Intercorrelations of Variables.

	*M*	*SD*	Skew	1	2	3	4	5	6	7	8
1 Dispositional LGO^a^T1	5.67	1.01	−.73	(.87)						
2 Workplace LGO^a^T1	3.82	0.88	−.80	.25**	(.90)					
3 Self-reference norm T1	3.60	0.89	−.24	.34**	.14	(.73)				
4 Constructive handling of errors T1	4.32	0.78	−.66	.00	.38**	−.12	(.82)				
5 Dispositional LGO^a^T2	5.51	0.95	−.61	.73**	.30**	.31**	.09	(.86)			
6 Workplace LGO^a^T2	3.77	0.84	−.64	.32**	.70**	.09	.31**	.34**	(.90)		
7 Self-reference norm T2	3.66	0.88	−.70	.29**	.21*	.47**	−.02	.33**	.20*	(.85)	
8 Constructive handling of errors T2	4.22	0.80	−.52	.03	.41**	−.11	.77**	.09	.43**	−.10	(.86)

*Note.* *N* = 144. T1 = measurement time point 1, T2 = measurement time point 2. Values in brackets denote reliability using Omega (ω_t_). *p < .05, **p < .01.

^a^LGO = learning goal orientation.

**Figure 1.**
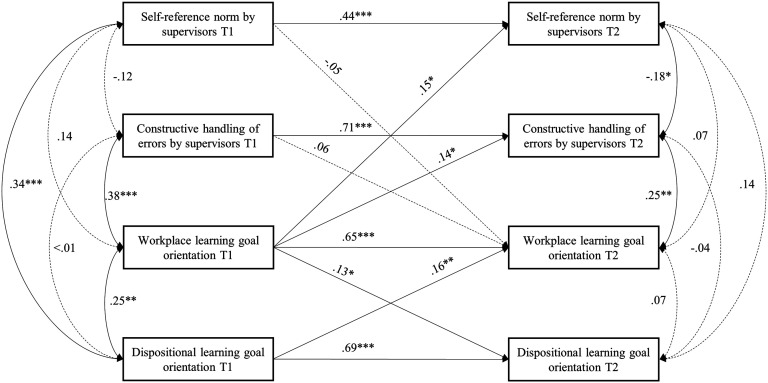
Standardized path coefficients for the reciprocal model (M2). *Notes.* T1 = measurement time point 1, T2 = measurement time point 2. *p < .05; **p < .01; ***p < .001.

Taking a look at the model comparisons (Table 2), the results indicated that our suggested model (M2) had a good model fit (Hu & Bentler, 1999) and was superior in terms of fit to the stability model (M1). The standardized solution for the final model is displayed in Figure 1. With regard to our hypotheses, first, the correlations between the variables from T1 and T2 were statistically significant and positive. Hence, the positive relationship of workplace learning goal orientation over time provides support for H1. Notably, the stability of this environmental variable is almost as high as for dispositional learning goal orientation. Second, we found support for several cross-lagged paths. Dispositional learning goal orientation at T1 was linked to changes in workplace learning goal orientation from T1 to T2 (supporting H2a). However, two of the expected paths in the reciprocal model were non-significant (the paths from the two appraisal facets at T1 to workplace learning goal orientation at T2). Hence, we found no support for lagged effects of the two supervisor’s appraisal behaviors on changes in workplace learning goal orientation from T1 to T2 (rejecting H3a). Nevertheless, the synchronous correlations at both measurement points indicated that employees who perceive their supervisor’s handling of errors as constructive, report at the same time also higher values of a workplace learning goal orientation. Third, with respect to reversed causation effects, workplace learning goal orientation at T1 was positively related to changes in dispositional learning goal orientation from T1 to T2 (supporting H2b) and to changes in the two appraisal facets (H3b). All cross-lagged paths identified in the model test represent large effects (Orth et al., 2022). In total, the highest amount of variance at T2 was explained for the constructive handling of errors (60%) by the variables included in the model, followed by dispositional learning goal orientation (54%), workplace learning goal orientation (52%), and the use of self-referenced norm by the supervisor (23%).^2^ In sum, although we did not find support for all our hypotheses, our results support the existence of reciprocal effects between dispositional, workplace learning goal orientation, and the appraisal behavior by the supervisor as perceived by the employees.

**Table 2.** Goodness-of-Fit Statistics Comparing the Stability Model to the Reciprocal Model.

Model	χ^2^	*df*	Model comparison: ∆χ^2^ (∆*df*)	NFI	IFI	CFI	RMSEA
M1 (Stability model)	28.65	12		.94	.96	.96	.10
M2 (Full reciprocal model)	5.13	6	23.52 (6)**	.99	1.0	1.0	.00

*Notes*. *N* = 144. χ^2^ = chi-square fit index; *df =* degrees of freedom; NFI = Normed Fit Index; IFI = Incremental Fit Index; CFI = Comparative-Fit-Index; RMSEA = Root-Mean-Square-Error-of-Approximation.

## Discussion

The promotion of a workplace oriented towards learning goals seems essential in today’s world of work based on the need for life-long learning. This is why we investigated the interplay of dispositional goal orientation and supervisor’s appraisal behavior at work that have the potential to stimulate such a learning goal-oriented work environment. Essentially extending prior research on workplace goal orientation and thereby contributing to theory development in this field (Schelp et al., 2022; Theis & Bipp, 2020; Van Dam, 2015), our findings shed light on the temporal stability of workplace learning goal orientation, their interplay with dispositional learning goal orientation, and the role of supervisor’s appraisal behavior.

First, our results indicate that workplace learning goal orientation is stable over a 5-week interval, at least to a certain degree. Overall, the stability coefficients obtained are comparable to the ones achieved for dispositional goal orientation (Payne et al., 2007), and we found stronger stability for a workplace learning goal orientation compared to research findings for the related construct of classroom goal structures (e.g., Bergsmann et al., 2013). This could be due to our rather short time interval between the two measurement points in our study, in which meaningful transitions that also might impact goal structures at work, like changes in the job or supervisor, were rather unlikely to occur. The fact that about half of the variance in our study could be explained by time effects nevertheless opens up possibilities for change or starting points for interventions aimed at supporting workplace learning goal orientation in practice.

Second, in line with the idea that goal orientations influence each other on different levels (e.g., individual, group, and organization; Chadwick & Raver, 2015), we found, on the one hand, that dispositional learning goal orientation is a predictor of workplace learning goal orientation over time. This finding is consistent with the reported meta-analytic correlation of trait learning goal orientation with the motivation to learn (Chung et al., 2022), or a positive feedback environment (Katz et al., 2021). With regard to possible determinants of workplace learning goal orientation, our findings highlight the role of dispositional learning goal orientation.

On the other hand, we found that a workplace that is directed towards personal growth and provides opportunities to learn, also seems to stimulate learning goal orientation in employees. Our finding supports the idea that goal structures at work can alter (rather stable) variables (Woods et al., 2013) and might stimulate future practical approaches to promote learning goal orientation among employees (Liu & Xiang, 2020; Wang et al., 2018). This relationship is in line with findings from the educational context (e.g. Bardach et al., 2020), and essentially extends empirical findings about potential antecedents of dispositional goal orientation (e.g. Payne et al., 2007). However, one has to keep in mind, that this relationship might also be explained by trait activation theory (Tett et al., 2021), according to which a workplace learning goal orientation might provide a trait-relevant situational cue for the expression of learning goal orientation of employees.

Third, our findings about the relationships between supervisor’s appraisal behavior and workplace learning goal orientation, provide evidence that core dimensions of the framework model of Benning et al. (2019) and findings from the educational domain can (at least partly) be transferred to the work context. Although in previous research a consistent positive correlation of appraisal that focuses on individual development and a learning goal-oriented environment with regard to contextual learning goal structure was found (e.g., Cecchini et al., 2014; Morgan et al., 2005), we obtained mixed support for such a relationship at work. While meta-analytic findings suggest that the perceived quality of the relationship with the supervisor is related to a positive feedback environment (Katz et al., 2021), and findings from the educational context indicate that changes in the behavior of teachers, for example, with regard to evaluation, are accompanied by changes in the perception of the goal structure by students (Janke et al., 2021), our results do not support a direct effect of appraisal behavior of supervisors on workplace learning goal orientation. While we build up our expectations on a well-researched theoretical framework to stimulate learning goals from the educational context (Ames, 1992a; Benning et al., 2019), using an intraindividual framework does not seem to play a key role for the perception of a learning goal structure within the workplace. And both appraisal facets investigated here were not related to workplace learning goal orientation over time.

Nevertheless, employees who consider their supervisor to handle errors in a constructive way also seem to perceive their environment as more learning goal-oriented given the intercorrelations we found at both points in time. This mirrors previous findings in the educational domain (Benning et al., 2019). In line with prior research that indicated that mastery goals might lead to a better quality of the exchange between leaders and employees (Janssen & Van Yperen, 2004), we found that a learning goal-oriented workplace also fosters in turn that supervisors pay attention to intraindividual standards or see mistakes as the opportunity to learn when evaluating performance or giving feedback. As supervisor support or feedback is an essential element for (in)formal learning at work (e.g., Decius et al., 2019), a workplace learning goal orientation might therefore indirectly support learning outcomes.

### Implications

In general, our findings provide much-needed insight into the construct of workplace learning goal orientation and therefore have important theoretical implications. Our results contribute to the research field by providing evidence to transfer previous findings about the stability, antecedents, or consequences of contextual goal structures from the educational to the work context. Together, we hope that our findings raise attention to and trigger more research about the construct of workplace goal orientation. For example, more research is needed to explicate process variables and identify moderators (e.g., feedback-seeking, self-efficacy, goal commitment; Breland & Donovan, 2005) for the effects of workplace goal orientation in practice, to fully integrate this construct into goal orientation theory (VandeWalle et al., 2019).

Also, our study has important consequences for organizational practice. Our findings point towards the positive, reciprocal effect between learning goal orientation at the dispositional and work environment level. Given their (separate) positive effects on organizational outcomes (e.g., Schelp et al., 2022; VandeWalle et al., 2019) it is helpful for practice to know that a learning goal orientation on various levels affects each other positively (see Chadwick & Raver, 2015). This finding implies a gain spiral, as such that enlarging one (or the perception of that) can also lead to positive effects for the other. So, to increase learning goals at work, organizations might consider dispositional goal orientation for the selection of employees (VandeWalle et al., 2019). Or, given that goals can be triggered by situational features (e.g. Bipp et al., 2021) or that the context in which individuals operate influences if and how we learn (Kyndt & Beausaert, 2017), we suggest adapting traditional performance management systems to integrate a focus on the development and learning of employees, rather than performance outcomes (alone) to realize a workplace learning goal orientation. Our results imply that supervisors’ appraisal behavior has the potential to affect a learning goal-oriented environment, particularly through the constructive handling of employees’ mistakes at work. Therefore, the implementation of workshops where the use and content of appraisal are thematized could be useful in practice.

However, given the lack of cross-lagged effects in our current study, organizations might also want to try other ways to stimulate a workplace learning goal orientation, for example, via challenging work (Van Dam, 2015). Furthermore, given prior findings showing the relevance of the TARGET or related frameworks to stimulate classroom goals (Ames, 1992a; Benning et al., 2019), successful interventions from the educational context might be adapted for the work setting and thoroughly tested in the future for their effectiveness. While we were successful in adapting and applying the measurement of (at least two) appraisal facets identified for teachers (Benning et al., 2019) to the work context, the fact that we did not find effects of these dimensions over time raises the need for future research to take a closer look also at other potential determinants of workplace learning goal orientation.

### Limitations and Future Research

Although our study has several strengths, for example, a repeated measurement design, a heterogeneous sample including employees with various backgrounds, and pre-tested measurement instruments, several limitations need to be borne in mind when interpreting the results. First, our data could reflect common-method biases because we assessed self-sourced and self-reported data by employees only (Podsakoff et al., 2003). Whereas goals are consistently defined and measured as subjective perceptions and cognitions (e.g., Van Dam, 2015; VandeWalle, 2001), the elicitation of supervisors’ appraisal behavior by employees’ perceptions might not reflect the view of the supervisors themselves. Thus, although we found support for reciprocal effects between the variables in our study, we cannot be sure if really the behavior of supervisors changed over time or the pure perception and evaluation of that by the employees. In particular, the chosen operationalization might form an alternative explanation for the overlap found between dispositional and workplace learning goal orientation or might be limited to the measures of dispositional learning goal orientation we used in the current study.

Second, the chosen time interval of 5 weeks between our measurement points might have been too short to cover meaningful reciprocal effects of the constructs investigated here. Although prior research in the area of goal orientation documented substantial variation of goal pursuit of teachers across a five-week span (Daumiller et al., 2023), we found rather high stabilities across this time period for (workplace and dispositional) learning goal orientations. Future research is therefore urged to systematically vary and investigate the stability (or change) of workplace goal orientation with different time frames.

Third, while we focused in our study on specific appraisal facets stemming from a long research tradition in the field of educational psychology, also other dimensions that are supposed to support a learning goal-oriented environment have been suggested (e.g., Lüftenegger et al., 2014). Even if appraisal (or evaluation) is assumed to be the most important dimension in this context, we found no evidence for the two facets investigated here for the work setting. Future research should take a closer look into the potential role of the third suggested dimension (effort-related feedback) and also other potentially relevant dimensions at work (like task or social characteristics; Benning et al., 2019). Therefore, more research is needed to examine the influence of other antecedents in line with the postulated framework of instructional dimension in longitudinal designs. This could provide a broader spectrum of potential intervention programs to foster a learning goal-oriented environment at work. Interestingly, in our study, the suggested two facets of the appraisal dimension of the framework of Benning et al. (2019) were largely unrelated (or partly even negatively related in the model test). This indicates that perceptions of self-reference norm and constructive error-handling may form rather distinct aspects underlying the appraisal dimension of the underlying framework. While their changes over time were similarly related to workplace learning goal orientation, this points to the merits of including further aspects of supervisor’s appraisal behavior in future research. Doing so would allow to follow up on the distinct aspects underlying this behavior and its factorial structure.

Fourth, although our diverse sample of employees might be an advantage of our study, the German-speaking participants who mainly worked in the private sector might form another limitation of the effects found.

## Conclusion

It is of utmost interest for organizational practice to understand how to effectively create and support work environments that engender learning. The workplace learning goal orientation construct provides an interesting starting point for establishing interventions to foster the motivation to learn and personnel development in practice, given that we found that it affects the perception of work environmental variables in terms of supervisor behavior. Together, the dispositional learning goal orientation of employees and afore-established workplace learning goal orientation play an important part in this as they influence each other positively, whereas the constructive handling of errors by supervisors seems to play an indirect role for the learning goal characteristics of the work environment.

## Data Availability

The data that support the findings of this study are available from the corresponding author upon request.

## Notes

1. In the present work, we consistently use the term *learning* instead of the frequently used term *mastery* to emphasize that we focus on goals that are based on intrapersonal competence development (see Daumiller et al. [2019] for a theoretical distinction of the different goal classes and the use of this label).

2. An additional model test carried out with participants age as control variable for dispositional learning goals at both time points, lead to comparable results.
